# Impact of maternal muscle strength on cesarean delivery outcomes: a comparative study of nulliparous women

**DOI:** 10.55730/1300-0144.5867

**Published:** 2024-07-02

**Authors:** Gülsemin ERTÜRK ÇELİK, Sezin ERTÜRK AKSAKAL, Yaprak ENGİN ÜSTÜN

**Affiliations:** 1 Division of Physical Medicine and Rehabilitation, Republic of Türkiye Ministry of Youth and Sports, Ankara, Turkiye; 2 Department of Obstetrics and Gynecology, University of Health Sciences, Etlik Zübeyde Hanım Women’s Health Training and Research Hospital, Ankara, Turkiye

**Keywords:** Maternal muscle mass, maternal muscle strength, handgrip test, myostatin, mode of delivery, cesarean section

## Abstract

**Background/aim:**

This study explored the correlation between maternal muscle mass and strength and the mode of delivery in childbirth. Specifically, it focused on full-term nulliparous pregnant women, analyzing ultrasonographic measurements of the quadriceps femoris muscle together with serum myostatin levels and muscle strength as determined by a handgrip test. The aim was to discern whether these factors could influence the likelihood of delivering vaginally or via cesarean section.

**Materials and methods:**

This study included 86 healthy nulliparous women at term, categorizing them into two groups based on their mode of delivery: vaginal delivery (58 women, Group 1) and cesarean section (28 women, Group 2). Comparative analyses of demographic information, delivery characteristics, ultrasonographic measurements of the quadriceps femoris, limb circumferences, handgrip strength, and serum myostatin concentrations were conducted.

**Results:**

The findings revealed that women in Group 1 had less gestational weight gain but greater handgrip strength compared to Group 2. Additionally, women who underwent cesarean section due to nonprogressive labor had greater arm and calf circumferences relative to those who had vaginal deliveries.

**Conclusion:**

The data of this study suggest a trend whereby lower maternal muscle strength and mass are associated with a decreased likelihood of vaginal delivery in pregnant women.

## 1. Introduction

Cesarean section, the most common significant surgical intervention worldwide, presents an intricate challenge in modern obstetrics [1]. In response to the escalating rates and inherent risks associated with this procedure, such as increased maternal morbidity and mortality, the American College of Obstetricians and Gynecologists (ACOG) together with the Society for Maternal-Fetal Medicine recently established comprehensive guidelines aimed at managing labor stages more effectively to curb the prevalence of primary cesarean sections [2]. These risks highlight the urgent need for innovative approaches to mitigate cesarean delivery rates compared to vaginal delivery [3].

The critical phase of labor, known as the second stage, encompasses the period from the cervix’s full dilation to the infant’s expulsion [4]. During this phase, the collaboration between uterine contractions and the mother’s abdominal muscle strength plays a pivotal role in facilitating the descent and expulsion of the fetus [5]. To assess maternal muscle strength, practitioners often employ methods such as the SARC-F questionnaire, designed for geriatric populations, and the direct measurement of handgrip strength [6]. For evaluating muscle mass, various diagnostic tools are available, including computed tomography (CT), magnetic resonance imaging (MRI), bioelectrical impedance analysis, ultrasonography, and dual X-ray absorptiometry (DXA), with the latter two being favored for pregnant women due to their cost-effectiveness and minimal radiation exposure [5].

Among the novel biomarkers for assessing muscle mass, myostatin, a member of the TGF-β superfamily, stands out. It acts as a crucial regulator of muscle physiology, inhibiting the proliferation and differentiation of satellite cells and thereby modulating muscle growth and maintaining its size within a physiological range [7]. It also plays a role in muscle-wasting conditions, being seen at lower concentrations in patients with sarcopenia [8].

This study explores the associations between muscle mass, determined by ultrasonographic assessments of the quadriceps femoris and myostatin levels, and muscle strength, determined via the handgrip test, in full-term nulliparous women undergoing different modes of delivery. It also addresses the relationship between maternal muscle characteristics and the necessity for cesarean section prompted by nonprogressive labor compared to standard vaginal delivery. This research could provide pivotal insights that challenge existing paradigms and foster a deeper understanding of the physiological underpinnings influencing delivery methods.

## 2. Materials and methods

This prospective cross-sectional study was approved by the Ethics Committee of Etlik Zübeyde Hanım Gynecology Training and Research Hospital (2020/82) and written informed consent was obtained from all patients.

Eighty-six healthy nulliparous full-term pregnant women between the ages of 20 and 40 admitted to the delivery room for vaginal (Group 1) or cesarean section (Group 2) delivery were included in the study during their follow-up. Mode of delivery, the indication for cesarean section, and the durations of the first, second, and third stages of labor in vaginal deliveries were investigated. Age, gravida, gestational week, body mass index (BMI; kg/m^2^), prepregnancy weight, gestational weight gain, newborn weight and sex, maternal complications (perineal laceration, postpartum hemorrhage, wound site infection), and neonatal complications (admission to neonatal intensive care unit, shoulder dystocia, neonatal death) were compared between the groups. Arm and calf circumferences, rectus muscle thickness measurements, handgrip strength, and myostatin levels were also compared. In subgroup analysis, we compared patients with cesarean delivery due to nonprogressive labor and those with vaginal delivery in terms of muscle strength and muscle mass parameters.

Power analysis revealed that to achieve effect size of d = 0.65 for muscle mass and muscle strength (90% power) with significance at the 5% level, a total of 85 participants would be required for vaginal and cesarean group comparisons [9].

Pregnant women under the age of 20 and over the age of 40; multiparous women; women at under 37 weeks and over 41 weeks of pregnancy; women with systemic diseases such as hypertension, diabetes, hypothyroidism, muscle disease, or neurological disease; cases involving fetal disease (oligohydramnios, polyhydramnios, intrauterine growth retardation, fetal anomaly, etc.); women who had undergone an operation that would affect muscle strength or muscle mass; and women with eating disorders were excluded from the study.

During electronic fetal monitoring, the development of at least one of the fetal heart rate traces defined as category 3 was considered as acute fetal distress according to the classification system published by the National Institute of Child Health and Human Development [10]. Cephalopelvic disproportion (mismatch between fetal head and maternal pelvis) was defined as the failure of labor to progress secondary to malposition of the fetal head (asynclitism, occiput posterior, mentum posterior, large fetus, etc.). Nonprogressive labor was defined as the absence of cervical change for at least 2 h when the cervix was dilated at least 4 cm or a second stage lasting longer than 2 h despite adequate uterine contractions, either spontaneously or with induction [4].

### 2.1. Muscle strength and muscle mass measurements

As previously defined by Lohman et al. [11], arm circumference was measured from the widest part of the left biceps muscle and calf circumference from the widest part of the left gastrocnemius muscle with a flexible plastic meter without putting pressure on the subcutaneous tissue. Rectus femoris muscle thickness, pennaculate angle, and muscle fiber length were measured by a single physician (S.E.A.) to evaluate muscle mass over the quadriceps femoris muscle during routine obstetric ultrasonography performed on admission to the delivery room, as defined by Martinoli [12]. The rectus femoris muscle was used for muscle mass measurement because, while there is no standardization for muscle mass measurement in pregnant women, physiological diastasis recti occurs in the rectus abdominis muscle and the rectus femoris is the most commonly used muscle for muscle mass measurement.

Handgrip strength was determined with a baseline hydraulic hand dynamometer (Jamar Hand Dynamometer, Irvington, NY, USA), which is routinely used around the world to measure muscle strength. In the sitting position, patients applied pressure by squeezing the dynamometer with their dominant hand in such a way that their hands were not supported by their bodies. Accordingly, muscle strength below 16 kg was defined as low grip strength in women [13].

### 2.2. Biomarker analysis

After obtaining informed consent from the patients included in the study, blood was collected in a 5-mL gel tube with a yellow cap with a serum separator together with routine blood tests on admission to the delivery room. Centrifugation was performed at 1000 × *g* for 15 min at 4 °C after waiting at least 30 min for the completion of the coagulation reaction. Serum samples were portioned into 2-mL polypropylene Eppendorf tubes. Samples were stored in a deep freezer at −80 °C until analysis. Analysis was performed by enzyme-linked immunosorbent assay (ELISA) method using the Elabscience E-EL-H5560 Human MSTN (Myostatin) ELISA Kit (BioCER Health Products and Devices Medical Company, Ankara, Türkiye).

### 2.3. Statistical analysis

Data were analyzed with IBM SPSS Statistics 23.0 for Windows (IBM Corp., Armonk, NY, USA). Mean, standard deviation, median, minimum, maximum, frequency, and ratio values were used for the descriptive statistics of the data. The distribution of variables was measured with the Kolmogorov–Smirnov test. Independent sample t-tests and Mann–Whitney U tests were used in the analysis of quantitative independent data. Chi-square tests were used in the analysis of qualitative independent data and Fisher tests were used when the chi-square test conditions were not met. Statistical significance was accepted at p < 0.05. Logistic regression analysis, in which BMI was added to the model as a covariate variable, was performed to evaluate whether the findings obtained were related to BMI, and it was observed that BMI did not significantly predict the mode of delivery and that findings obtained for other parameters did not change at the specified level of significance.

## 3. Results

A total of 86 patients were included in the study. During the follow-up of the pregnant women admitted to the delivery room, 58 (67.5%) delivered vaginally and 28 (32.5%) delivered by cesarean section. The mean age, gestational week, gravida, BMI, and abortion numbers of both groups were similar (p > 0.05) (Table 1). The mean prepregnancy weight was not different between the groups. Weight gained during pregnancy was significantly higher in the group that delivered by cesarean section (15.8 ± 6.6 kg vs. 11.8 ± 5.3 kg; p = 0.004) (Table 1).

The indications for cesarean section were cephalopelvic disproportion (n = 12, 42.9%), nonprogressive labor (n = 11, 39.3%), and fetal distress (n = 5, 17.9%).

While the mean duration of the first stage of labor was 6.8 ± 2.8 h (2.1–15.0 h), the duration of the second stage was 50.5 ± 22.3 min (12–126 min) and the duration of the third stage was 11.8 ± 3.4 min (5.0–20.0 min) among patients who had vaginal delivery.

When the groups were evaluated in terms of maternal muscle strength, muscle mass, and myostatin levels, the mean myostatin level, maternal arm and calf circumferences, and rectus femoris thickness were similar between the groups (p > 0.05). Handgrip strength was found to be significantly higher in patients who had vaginal delivery (24.1 ± 4.2 kg) compared to those who delivered by cesarean section (23.0 ± 3.09 kg) (p = 0.020) (Table 2).

In addition, myostatin, arm circumference, calf circumference, rectus femoris thickness, and handgrip strength were also compared between patients who had cesarean section due to nonprogressive labor and those who delivered vaginally. It was found that the arm circumference (median = 34.00 cm) of the patients in the nonprogressive labor group was significantly higher than that of the patients who had vaginal delivery (median = 30.50 cm) (p = 0.037). The calf circumference of the patients in the nonprogressive labor group (median = 68.00 cm) was also found to be significantly higher than that of the patients who had vaginal delivery (median = 61.00 cm) (U = 189.500, z = −2.128, p = 0.033). There was no significant difference between the patients who underwent cesarean section due to nonprogressive labor and the patients who had vaginal delivery in terms of myostatin, rectus thickness, or handgrip strength (p > 0.05) (Table 3).

## 4. Discussion

This study is the first to analyze maternal muscle strength, muscle mass, and myostatin levels according to mode of delivery in nulliparous women at term, a decisive issue often overlooked in obstetrics research. The findings revealed that lower handgrip strength and higher weight gain during pregnancy are significantly associated with an increased likelihood of cesarean delivery, particularly in cases arising from nonprogressive labor. This trend, consistent with broader maternal health challenges, mirrors the patterns observed in other demographic groups and aligns with the established associations of higher arm and calf circumferences with cesarean interventions.

Our study’s focus on the rectus femoris muscle, frequently utilized in muscle mass assessments, is significant in understanding maternal physiology. The finding that maternal muscle strength and mass might not significantly differentiate between vaginal and cesarean deliveries may open new avenues of research in obstetric science. Our pioneering measurement of muscle strength in pregnant women using the Jamar hand dynamometer, revealing a marked decrease in those who underwent cesarean section, suggests that abdominal and myometrial muscle strength could be instrumental in natural childbirth.

In the study conducted by Mohammadian et al. [14], the mean muscle strength of the dominant hand as measured by Jamar hand dynamometer was reported as 26.5 ± 6.1 kg in healthy women, 28.5 ± 6.2 in the age range of 20–24 years, and 29.9 ± 4.6 kg in the age range of 25–29 years. In the metaanalysis published by Bohannon et al. [15], the mean right- and left-hand muscle strength was 30.6 (26.7–34.4) kg and 27.9 (23.1–32.6) kg for women aged 20–24 years while the mean right- and left-hand muscle strength was 33.8 (29.5–38.1) kg and 30.8 (27.2–34.5) kg for the age group of 25–29, respectively. In the healthy Swiss population, muscle strength as measured with the Jamar hand dynamometer was found to be 33.4 ± 5.4 kg in healthy women aged 20–24 years and 34.3 ± 5.7 kg in the age range of 25–29 [16]. In our study, the mean age was 22.9 ± 4.2 years and mean muscle strength was 25.1 ± 4.2 kg in pregnant women who had vaginal delivery, while mean age was 25.3 ± 5.8 years and mean muscle strength was 23.0 ± 3.0 kg in pregnant women who delivered by cesarean section. The lower mean value of muscle strength in both groups of patients compared to findings reported in the literature could result from the fact that our patients were in the last trimester with increased intraabdominal pressure and the onset of labor.

The second stage of labor is the period between full cervical dilatation and the delivery of the fetus. In the second stage, the descent of the fetus puts pressure on the bladder and rectum and creates a bear-down reflex in the mother. The straining reflex, on the other hand, helps the mother support uterine contractions by using her abdominal and respiratory muscles to push the fetus downwards and complete the fetal cardinal movements [17]. There are few studies in the literature evaluating maternal muscle strength and muscle mass during pregnancy. It has been found that rectus abdominal muscle thickness decreases during pregnancy [18]. Kawanabe et al. [19] investigated the relationship between total arm and leg muscle mass and insulin resistance and found that the ratio of skeletal muscle mass to fat mass was positively correlated with insulin resistance. On the other hand, Çintesun et al. [20] investigated the physiological development of diastasis recti in the rectus abdominis muscle during pregnancy and the duration of the second stage of labor and found a significant relationship between the distance between the rectus muscles at the xiphoid level and the duration of the second stage of labor in primiparous patients. Since diastasis recti develops physiologically in the rectus abdominis muscle during pregnancy and the rectus femoris muscle is used most frequently in muscle mass measurements, the rectus femoris muscle was used in our study. Our study investigated whether maternal muscle strength and muscle mass are factors in cesarean delivery, but no difference was found between vaginal and cesarean delivery patients. To our knowledge, this is the first study to measure muscle strength in pregnant women. Muscle strength measurements obtained with a Jamar hand dynamometer were found to be significantly lower in patients who underwent cesarean section. Women with less muscle strength may not deliver vaginally because their abdominal and myometrial muscle strength is also lower.

It is known that maternal obesity increases the risk of many intrapartum and postpartum complications such as induction, macrosomia, shoulder dystocia, and cesarean section [1,21]. The rates of primary cesarean section and emergency cesarean section were found to be significantly increased in obese primiparous women [22,23]. In our study, nulliparous full-term pregnant women were included, and the weight gained during pregnancy was found to be higher and muscle strength was lower in those who underwent cesarean section compared to the patients who had vaginal delivery. If these results are supported by further studies together with exercise and diet aimed at increasing maternal muscle strength, the vaginal birth rate will increase and cesarean rates might decrease accordingly.

The literature reports that cesarean delivery has an average complication rate 3 times higher than that of vaginal delivery [24]. While no difference was found in terms of maternal and neonatal complications in our study, the 1-min and 5-min APGAR scores were found to be significantly lower among cases of cesarean section delivery. These low APGAR scores may have been due to the small number of patients, the fact that only primary cesarean section cases were evaluated, or the fact that 17% of the cesarean deliveries were performed due to fetal distress.

The important feature of this study is that it is the first study to evaluate muscle strength, muscle mass, and myostatin levels in full-term pregnant women. However, this study is not without its limitations. First, the sample size was adequate to demonstrate trends but may lack the statistical power required to generalize the outcomes to a broader population. The absence of a more diverse demographic profile also narrows the scope of the results, potentially limiting their applicability across different ethnicities and socioeconomic backgrounds. Furthermore, the exclusive reliance on the Jamar hand dynamometer for assessing muscle strength and the failure to utilize comprehensive diagnostic tools like MRI to evaluate total muscle mass could constrain the depth of our conclusions. Such methods might have provided a more holistic understanding of muscle status and its impact on labor outcomes. Additionally, the cross-sectional nature of the study precludes the establishment of a causal relationship among muscle strength, muscle mass, and mode of delivery. It is also important to note that this study focused on nulliparous women at term; thus, the findings may not reflect the complexities of multiparous women or those with different pregnancy courses. Finally, environmental and lifestyle factors that could influence muscle strength and mass, such as physical activity levels and nutritional status, were not exhaustively controlled or measured. These limitations highlight the need for future research to incorporate longitudinal designs, larger and more varied cohorts, and comprehensive muscle assessment techniques to build upon the findings presented here.

Maternal expulsive power is one of the most important factors leading to the birth of a fetus with accompanying uterine contractions, especially in the second stage of labor. In our study, we found low handgrip strength in patients who underwent cesarean section and high arm and calf circumferences in patients who underwent cesarean section due to nonprogressive labor. Based on these findings, it can be said that pregnant patients with low muscle strength and muscle mass are less likely to deliver vaginally. It may be possible to reduce the rate of primary cesarean section with studies aimed at increasing muscle strength and muscle mass (via exercise, diet, etc.) during pregnancy. Large series of randomized controlled studies are needed to reach a definite conclusion.

This study’s strength lies in its novelty and its potential to guide obstetric practices. Our findings tentatively suggest a correlation between diminished maternal muscle strength and cesarean delivery with a need for more expansive randomized controlled trials to confirm these observations. Such knowledge could refine our understanding of labor mechanics and potentially revolutionize our approach to reducing primary cesarean section rates, thereby enhancing the wellbeing of mothers and neonates alike.

In conclusion, this pioneering study has shed critical light on the underexplored interplay among maternal muscle strength, muscle mass, and mode of delivery. The finding that nulliparous women at term with lower handgrip strength and higher gestational weight gain are more likely to undergo cesarean delivery, particularly due to nonprogressive labor, suggests new avenues of research for obstetric interventions. This study may provoke compelling dialogue about the potential of targeted muscular conditioning to decrease cesarean rates, which could hold profound implications for maternal health strategies. By highlighting the importance of maternal physical conditioning, this research stands not only as a testament to the intricate physiology governing childbirth but also as a beacon guiding future clinical practices towards more natural delivery processes. It emphasizes the importance of maternal fitness in labor and delivery, a field of research ripe for further scholarly exploration and clinical innovation. Thus, this study is not just a collection of data; it is a call to action for a paradigm shift in the care and preparation of expectant mothers for the pivotal act of childbirth.

## Figures and Tables

**Table 1:** Comparison of demographic characteristics, neonatal characteristics, and maternal and neonatal complications in terms of delivery modes.

	Group 1 (vaginal delivery) n = 58	Group 2 (cesarean delivery) n = 28	p

Age *	22.9 ± 4.2	25.3 ± 5.8	0.089 m

Gravida˚	1 (1–2)	1 (1–2)	0.774 m

Abortus˚	0 (0–1)	0 (0–1)	0.756 m

Gestational week *	38.9 ± 1.2	38.6 ± 1.3	0.228 m

BMI (kg/m^2^)*	27.3 ± 3.9	27.4 ± 4.5	*0.331* t

Prepregnancy weight (kg)*	60.7 ± 9.3	62.2 ± 11.9	0.512 t

Gestational weight gain (kg)*	11.8 ± 5.3	15.8 ± 6.6	***0.004*** t

Neonatal Weight (gr)*	3291 ± 355	3374 ± 526	0.796 m

Neonatal sex*			
Girl	29 (50.0%)	12 (42.9%)	0.534 X^2^
Boy	29 (50.0%)	16 (57.1%)	

APGAR 1*	9.0 ± 0.0	8.8 ± 0.8	***0.041*** m

APGAR 5*	10.0 ± 0.0	9.8 ± 0.6	***0.012*** m

PrepartumHb (gr/dL)*	11.6 ± 1.6	11.7 ± 1.4	0.716 m

Postpartum Hb(gr/dL)*	10.6 ± 1.7	10.4 ± 1.3	0.326 m

Maternal complicationˠ	6 (10.3%)	5 (17.9%)	0.328 X^2^

Neonatal complicationˠ	0 (0.0%)	1 (3.6%)	0.326 X^2^

t **t-test /**

m **Mann-Whitney U test**.

X^2^ **Chi-square test**. BMI: Body mass index, *p*-values with statistical significance (p < 0.05) are shown in bold

˚ Data presented as median (min–max )

* Data presented as mean ± SD

ˠ , Data presented as n (%).

**Table 2:** Comparison of maternal muscle strength and muscle mass in patients with vaginal and cesarean delivery.

	Group 1 (vaginal delivery) n = 58	Group 2 (cesarean delivery) n = 28	p
Myostatin (ng/mL)	3.5 ± 3.2	3.5 ± 2.9	0.962 m
Arm circumference (cm)	29.8 ± 3.9	33.4 ± 7.5	0.080 m
Calf circumference (cm)	62.2 ± 7.8	65.4 ± 10.0	0.248 m
Rectus thickness (mm)	18.0 ± 2.3	19.0 ± 3.7	0.380 m
Handgrip strength (kg)	25.1 ± 4.2	23.0 ± 3.0	**0.020** m

m **Mann-Whitney U** test, Data presented as mean ± SD. P-values with statistical significance (p < 0.05) are shown in bold.

**Table 3:** Comparison of myostatin, arm circumference, calf circumference, rectus femoris thickness, and handgrip strength between patients undergoing cesarean delivery due to nonprogressive labor and those with vaginal delivery.

	Cesarean delivery due to nonprogressive labor (n = 11)	Vaginal delivery (n = 58)	p
Myostatin (ng/mL)	2.31 ± 1.75	3.55 ± 3.17	0.204^m^
Arm circumference (cm)	35.18 ± 7.80	29.84 ± 3.87	**0.037** m
Calf circumference (cm)	68.55 ± 8.97	62.21 ± 7.84	**0.033** m
Rectus thickness (mm)	18.86 ± 3.41	18.01 ± 2.36	0.731^m^
Handgrip strength (kg)	24.04 ± 2.91	24.00 ± 4.26	0.442^t^

t **t-test /**

m **Mann-Whitney U test**.

Data presented as mean ± SD. P values with statistical significance (p < 0.05) are shown in bold.
